# Creativity beyond the isolated mind: toward a biopsychosocial and sociotechnical perspective

**DOI:** 10.3389/fcogn.2026.1893765

**Published:** 2026-09-28

**Authors:** Maria Adriana Neroni

**Affiliations:** Department of Communication, Education and Psychology Studies, Suor Orsola Benincasa University, Naples, Italy

**Keywords:** biopsychosocial perspective, creative cognition, creativity, distributed cognition, sociotechnical systems

## Abstract

Creativity has traditionally been studied at different levels of analysis, focusing on neural mechanisms, cognitive abilities, individual characteristics, or environmental influences. Although each approach has provided important insights, none alone fully explains why the same factors may support creativity under some conditions but constrain it under others, or why creative potential is expressed differently across individuals, domains, developmental stages, and sociocultural contexts. Building on biopsychosocial, systemic, sociocultural, and interactionist approaches, this Perspective conceptualizes creativity as a multilevel, developmentally situated, and sociotechnically mediated phenomenon emerging from interactions among biological, psychological, sociocultural, and technological processes. The central proposition is that no single factor is inherently creative or uncreative; its contribution depends on how it interacts with other factors under particular conditions. The framework therefore shifts the unit of explanation from isolated determinants to changing configurations of relationships across levels. Four mechanisms, i.e., constraint, affordance, regulation, and feedback/selection, describe how these relationships may develop over time and generate testable predictions concerning differences across individuals, stages of the creative process, developmental periods, and levels of analysis. Evidence from neuroscience, psychology, sociocultural research, education, organizational studies, and generative artificial intelligence illustrates how apparently contradictory findings may partly reflect these differences. Methodologically, the framework calls for greater conceptual and measurement specificity and for ecological, longitudinal, multilevel, and interdisciplinary research. Rather than asking which factors universally increase creativity, it asks for whom, under which conditions, at which stage and level, and over what timescale particular factors support or constrain creative activity.

## 1 Introduction

Creativity has been studied from multiple levels of analysis. Biological and neuroscientific approaches have examined neural mechanisms and biological predispositions; psychological research has focused on cognitive abilities, personality, motivation, and emotion; and sociocultural perspectives have emphasized the relational and institutional conditions that shape creative activity. Each tradition has provided important insights, yet none alone fully explains why creativity emerges, why it varies across individuals, domains, developmental periods, and cultures, or why creative potential does not always translate into creative behavior or socially recognized achievement (Abraham, 2022; Benedek, 2024). Increasingly, research points to the importance of context and interaction, challenging explanations of creativity as either a stable individual property or the consequence of a single causal factor (Glăveanu, 2020; Tromp and Sternberg, 2022).

Building on a biopsychosocial perspective (Bolton and Gillett, 2019), this article conceptualizes creativity as a multilevel, developmentally situated, and sociotechnically mediated phenomenon. However, the proposed framework goes beyond treating biological, psychological, and social influences as separate factors whose contributions can simply be combined. Instead, it views creativity as emerging from changing relationships among biological, psychological, developmental, sociocultural, and technological processes. A factor such as cognitive flexibility, intrinsic motivation, social support, domain expertise, or technological assistance may support creativity under some conditions while having little effect or even becoming constraining under others.

This emphasis on interaction builds on, but also extends, existing systemic, sociocultural, and interactionist approaches. Csikszentmihalyi's (1999) systems model shifted attention away from the isolated creator by locating creativity in the relationship among person, domain, and field and highlighting the role of social recognition. Glăveanu's (2013); Glăveanu (2020) sociocultural approach similarly challenged the separation between individual and society, as well as between mind and material environment, by emphasizing relations among actors, actions, artifacts, audiences, and affordances. More recently, the Person × Task × Situation framework has described creativity as emerging from interactions whose effects vary across people, tasks, situations, and the lifespan (Tromp and Sternberg, 2022).

The present framework shares this relational view but extends it across levels of analysis. It considers how psychological and sociocultural processes are themselves shaped by embodied and neurobiological conditions, developmental trajectories, and increasingly by technological systems. Its central proposition is therefore not simply that creativity has multiple determinants. Rather, the relevant unit of explanation is the changing configuration of relationships among these determinants over time. This distinction is important because the same factor may play different roles depending on the individual, the task, the stage of the creative process, and the broader social or technological environment.

The heterogeneity of findings across creativity research provides one reason to adopt this perspective. In neuroscience, for example, the shift from localisationist explanations toward network-based models represented an important theoretical advance (Beaty et al., 2016, 2019). However, recent critiques caution against replacing one form of reductionism with another. Abraham (2025a) highlights substantial differences across creativity studies in tasks, scoring methods, neural findings, and individual strategies, questioning whether findings concerning the default mode network can be generalized to creativity as a whole. Dietrich (2024) raises a broader concern, arguing that creativity should not be treated as a single cognitive faculty with a dedicated neural substrate. Together, these critiques suggest that neural systems may contribute differently depending on the form of creative activity, the cognitive processes required, individual characteristics, and the context in which creativity occurs.

A similar pattern emerges in psychological and sociocultural research. Divergent thinking, executive control, motivation, emotion, personality, domain knowledge, and environmental support have all been associated with creativity, but their effects vary across tasks and contexts. Abraham (2022), for example, emphasizes that context matters throughout the generation, development, and reception of creative ideas and differs substantially across creative domains. Such variability need not simply indicate weakness or inconsistency in creativity research. It may instead reflect an important feature of creativity itself: a particular factor's contribution depends on how it interacts with other individual, task-related, developmental, and sociocultural conditions.

From this perspective, creativity emerges through the interplay of several systems. Biological processes provide the embodied conditions for creative cognition; psychological processes shape how ideas are generated, evaluated, and transformed; developmental experiences influence the knowledge, habits, and strategies that individuals bring to creative activity; and social and cultural environments affect which possibilities become meaningful, valued, and ultimately recognized. These dimensions continually influence one another. As a result, the same individual may display different forms or degrees of creativity across life stages, domains, tasks, and environments as the conditions supporting creative activity change.

Technological environments have become an increasingly important part of this picture. Platforms, recommendation systems, algorithms, and generative artificial intelligence influence not only access to information but also how ideas are generated, selected, circulated, and evaluated. Technology therefore cannot always be treated as a neutral tool external to the creative process; it can actively shape the possibilities available to creators. Recent research on generative AI provides a particularly clear illustration of this complexity, showing that technological assistance can affect different components and levels of creative activity in different ways (e.g., Chen and Chan, 2024; Doshi and Hauser, 2024; McGuire et al., 2024). Rather than asking simply whether technology, or any other factor, “increases creativity,” it may therefore be more informative to ask for whom it is beneficial, under which conditions, at which stage and level of creative activity, and over what period of time.

Similar caution is needed when defining and measuring creativity. The widely used standard definition describes creative products as both original and effective or useful (Runco and Jaeger, 2012). Although this definition has provided an important common reference point, it remains debatable. Abraham (2025b), for example, argues that defining creativity mainly through novelty and usefulness emphasizes external evaluation more than the creator's experience and the creative process itself. More generally, social, cultural, historical, and technological contexts shape judgments of originality, appropriateness, and value. A scientific theory, artistic work, technological invention, or everyday solution does not acquire creative significance in isolation; its meaning and value depend partly on the practices, standards, and communities within which it is produced and recognized (Csikszentmihalyi, 1999; Glăveanu, 2020).

In light of these considerations, the aim of this Perspective is neither to replace single-factor explanations with an exhaustive list of determinants nor to provide a comprehensive review of creativity research. Instead, it proposes a shift in explanatory focus: from asking which individual factors produce creativity to examining how different factors work together and acquire significance under particular conditions. The paper also considers the theoretical and methodological implications of this shift.

For analytical clarity, the following sections examine biological, psychological, sociocultural, and technological dimensions separately. This separation is primarily analytical, as the paper treats these dimensions as interdependent components of a changing creative system. The central claim is that creativity can be better understood by shifting the unit of explanation from isolated determinants to the evolving relationships among embodied, cognitive, developmental, relational, cultural, and technological processes that enable or constrain creative activity.

## 2 The creative body: neural networks, biological predispositions, and developmental trajectories

Biological approaches to creativity have changed substantially over the past few decades. Earlier research often sought to identify specific brain regions, biological predispositions, or hereditary characteristics associated with creative ability. Although this work provided important insights, it also encouraged the idea that creativity might ultimately be traced to a relatively stable biological substrate. More recent research offers a more complex picture. Biological processes are increasingly understood as providing conditions and constraints that interact with cognitive, developmental, and environmental factors rather than determining creativity directly. From the perspective proposed here, biology matters not because it contains creativity, but because it shapes the conditions under which creative activity becomes possible.

One important development has been the shift from localisationist explanations toward network-based approaches. Rather than searching for a discrete “creative brain area,” researchers have examined how creative cognition draws on distributed neural systems involved in associative processing, memory, internally directed thought, attentional control, and executive regulation (Beaty et al., 2016, 2019; Benedek and Fink, 2019). Particular attention has focused on interactions between the default mode network (DMN) and the executive control network (ECN). The DMN supports processes such as internally directed thought, episodic and autobiographical memory, mental simulation, and spontaneous associative processing, whereas the ECN contributes to working memory, attentional control, goal maintenance, and evaluating emerging ideas (Beaty et al., 2016; Benedek and Fink, 2019). The salience network has also been proposed to help coordinate shifts between internally and externally oriented processing in response to changing demands (Menon, 2015). On this account, creative cognition does not reside within any single network but may depend on flexible coordination among processes that generate, monitor, and evaluate ideas.

Empirical studies have provided support for this view. Creative idea generation, particularly during divergent-thinking tasks, has been associated with functional coupling between default-mode and executive systems (Beaty et al., 2015, 2016, 2019). These findings have contributed to the influential proposal that creative thinking involves cooperation between networks supporting spontaneous or internally generated thought and those involved in cognitive control. However, this account should not be taken to mean that the neural basis of creativity is now well established. Abraham (2025a) cautions against generalizing findings obtained from specific creative-thinking tasks to creativity as a whole. Studies differ substantially in the tasks they use, how they score creativity, and the neural patterns they report, while individual differences in how people approach creative tasks are often overlooked. Evidence of DMN–ECN involvement in divergent thinking therefore does not necessarily imply a general neural system for creativity. These networks may instead support particular cognitive processes whose relevance varies across tasks, individuals, and forms of creative activity (Abraham, 2025a).

Dietrich (2024) raises a broader concern about the neuroscience of creativity. He argues that research often treats creativity as a single cognitive ability and, consequently, searches for a specific neural system responsible for it. Yet creative activities can involve very different combinations of cognitive, emotional, perceptual, motor, and domain-specific processes. Creativity is therefore unlikely to depend on a single dedicated neural mechanism. Different creative activities may instead recruit different combinations of neural and cognitive processes that also support many other forms of behavior (Dietrich, 2024). This view is consistent with the present framework because it shifts the focus from identifying *where* creativity is located in the brain to understanding *which* processes contribute to a particular creative activity, for a particular individual, and under particular conditions.

These considerations also call for caution in interpreting neuroimaging findings. Much of the neuroscience of creativity has relied on divergent-thinking tasks, particularly variants of the Alternative Uses Task, because they offer experimentally manageable ways to study idea generation. Such paradigms have yielded valuable evidence about the neural processes associated with producing multiple or unusual responses (Beaty et al., 2015, 2016). However, divergent thinking captures only one aspect of creative cognition and should not be treated as equivalent to creativity more broadly (Benedek, 2024; Dietrich, 2024). Neural patterns observed during these tasks are therefore better interpreted as correlates of particular forms of ideational processing than as neural signatures of creativity itself. This distinction is especially important when moving from brief laboratory tasks to real-world creative activities, which may unfold over long periods and involve expertise, material interaction, emotion, collaboration, evaluation, and social recognition (Abraham, 2022; Dietrich, 2024).

Evidence from neurological lesions points in a similar direction. Lesions affecting different brain regions can alter aspects of creative performance such as fluency, flexibility, and originality, while anatomically distinct lesion sites may be connected to common functional circuits (Shofty et al., 2022). These findings do not identify a single creative center. Instead, they suggest that different components of creative performance depend on distributed neural systems and that the consequences of neural disruption vary by the process and form of creativity examined.

Genetic research also provides little support for simple biological explanations. Studies have reported associations between creative performance and genetic variation related to dopaminergic functioning, including interactions involving the DAT and COMT genes (Zabelina et al., 2016). However, creativity is a complex behavioral phenotype and is unlikely to map directly onto individual genetic variants. Genetic influences are more plausibly understood as indirect and probabilistic, operating through characteristics such as cognitive functioning, temperament, reward sensitivity, and neural plasticity. Their expression also depends on developmental and environmental conditions. A biological predisposition associated with exploratory behavior, for example, cannot be assumed to enhance creativity independently of learning opportunities, domain expertise, motivation, cultural expectations, or the environment in which exploration takes place. Genetic influences should therefore be considered part of a broader developmental system rather than a biological starting point that determines later creative ability.

A developmental perspective further challenges the idea that creativity is biologically fixed. Lifespan research distinguishes among creative potential, everyday creative behavior, domain-specific performance, and recognized creative achievement, all of which can follow different developmental trajectories (Bornstein, 2021; Benedek, 2024). Neural maturation, cognitive control, knowledge, motivation, emotional regulation, and physical capacities change alongside changes in education, social roles, access to resources, and opportunities for creative participation. There is therefore no single developmental trajectory of creativity. Instead, the resources that support different forms of creative activity change across the lifespan.

Childhood provides a clear example. Neural plasticity and exploratory behavior may favor learning, experimentation, and the formation of unusual associations, but these characteristics should not be equated with creativity itself. Children also have less accumulated domain knowledge and experience, and whether their exploratory behavior results in outcomes judged creative depends on the task, developmental stage, educational environment, available materials, and the criteria used to evaluate creativity (Bornstein, 2021). Later adulthood presents a different configuration of resources. Some cognitive abilities, such as processing speed, may decline with age, whereas domain knowledge, semantic knowledge, expertise, and accumulated experience can continue to support creative activity (Bornstein, 2021). Age should therefore not be expected to produce a uniform increase or decline in creativity; instead, it changes the combination of resources through which different forms of creativity can emerge.

Development also illustrates why biological and environmental processes cannot be cleanly separated. Neural systems change through experience, while the experiences available to individuals depend on their capacities, interests, relationships, opportunities, and environments. Repeated engagement in artistic, scientific, technical, or everyday creative activities can alter knowledge, strategies, and patterns of cognitive processing, while access to these experiences is shaped by educational opportunities, socioeconomic resources, cultural expectations, interpersonal relationships, and technological infrastructures. Development is therefore not simply a biological process later influenced by the environment; it emerges through continuous interactions between individuals and their environments (Bornstein, 2021).

Research on sex and gender provides another example of why biological variables should not be interpreted independently of context. Although sex-related differences have been reported for particular creative domains and measures, evidence for general differences in creative ability remains inconsistent. A recent systematic review and meta-analysis found only minimal average gender differences in creative ability and concluded that differences in variability cannot explain the much larger gender disparities observed in eminent creative achievement (Taylor et al., 2024). Another large meta-analysis showed that gender differences in creativity vary by the psychological processes examined and task characteristics, including whether perspective-taking and usefulness are emphasized (Kim et al., 2024). These findings suggest that observed differences cannot be explained solely by biological sex.

Disparities in creative achievement may also reflect differences in educational opportunities, institutional barriers, gender expectations, professional networks, encouragement, access to resources, and systems of cultural recognition. Biological characteristics may interact with these factors, but group differences in creative outcomes cannot be assumed to originate from biological sex itself. Sex and gender therefore illustrate the broader argument developed here: the significance of a biological characteristic depends partly on the developmental, psychological, social, and cultural conditions in which it operates.

Overall, biological research does not identify a single neural, genetic, or developmental foundation for creativity. Instead, it points to multiple biological processes whose contribution varies across tasks, individuals, developmental stages, and contexts. The heterogeneity of these findings should not necessarily be regarded as a problem to be solved by searching for a universal biological marker of creativity. Instead, it may reflect that creativity can emerge through different combinations of biological and cognitive processes. Within the framework proposed here, biological systems provide changing embodied possibilities and constraints that experience and context continuously shape. Creativity therefore emerges not from biology alone, but from the interaction between biological capacities and the cognitive, developmental, relational, cultural, and technological environments in which they are expressed.

## 3 The creative mind: regulation, ideation, motivation, and emotion

While biological approaches focus on the embodied conditions that make creative activity possible, psychological research examines how people generate, evaluate, select, and develop ideas. Creativity has been associated with a wide range of cognitive, motivational, emotional, metacognitive, and personality-related factors. However, an association with creativity does not mean that any of these factors has a stable or independent effect. The same psychological process may support creativity in one situation and constrain it in another, depending on the task, the stage of the creative process, individual characteristics, and the broader context. Psychological factors are therefore better understood as resources whose contribution changes according to the conditions in which they operate.

A central idea in contemporary research is that creativity involves both generative and regulatory processes. Creative ideation draws on memory retrieval, associative exploration, semantic recombination, and imagination, but ideas must also be monitored, evaluated, selected, and adapted to current goals and constraints (Benedek et al., 2023; Benedek and Fink, 2019). Research on memory, for example, suggests that creative ideas arise not only from spontaneous associations but also from goal-directed retrieval and the flexible recombination of existing knowledge (Benedek et al., 2023). Creativity may therefore require different balances between spontaneous and controlled processing at different stages rather than consistently high or low levels of cognitive control.

Executive functions provide a clear example. Working memory, inhibition, attentional control, and cognitive flexibility can help individuals maintain goals, suppress dominant responses, shift between perspectives, and adapt strategies when current approaches are unsuccessful (Benedek et al., 2014; Benedek and Fink, 2019). Yet stronger executive control is not always beneficial. Control may be particularly useful when a task requires evaluation, refinement, or adherence to constraints, whereas less constrained processing may sometimes facilitate access to unusual associations during idea generation (Beaty et al., 2016; Benedek and Fink, 2019). The relationship between control and creativity is therefore better understood as adaptive regulation: what matters is not maximizing control or flexibility, but using these processes in ways that fit the demands and stage of the creative activity.

Cognitive flexibility illustrates this point particularly well. Shifting perspectives or moving between conceptual categories can help restructure an ineffective representation and open alternative routes to a solution. However, constant shifting may become counterproductive when creative work requires sustained elaboration or commitment to a promising idea. Inhibition can similarly help suppress dominant but unproductive responses, while excessive control may restrict access to less conventional possibilities (Benedek et al., 2014; Benedek and Fink, 2019). Apparently conflicting findings on the role of cognitive control may therefore reflect differences in tasks, domains, expertise, or the stage and component of creativity being assessed rather than a simple opposition between controlled and spontaneous cognition.

Metacognition offers a useful way to understand how these processes are coordinated. Lebuda and Benedek (2023) distinguish between the cognitive processes involved in performing a creative task and the metacognitive processes used to monitor and regulate performance. Creative activity requires not only generating ideas, but also assessing progress, evaluating possible responses, selecting strategies, and deciding whether to continue exploring or change direction. Metacognitive feelings can also indicate whether ideation is progressing effectively and whether a current strategy should be maintained or revised (Puente-Díaz, 2023). Generation and evaluation are therefore not simply separate stages. Monitoring can influence subsequent idea generation, while new ideas can change evaluation criteria and strategic choices.

This perspective also complicates the traditional distinction between divergent and convergent thinking. Divergent-thinking tasks have played a major role in creativity research because they offer manageable ways to assess fluency, flexibility, and originality. They remain useful indicators of aspects of creative potential, and recent evidence shows meaningful associations between divergent-thinking performance and creative activity and accomplishment (Runco and Alabbasi, 2024). However, divergent thinking is not equivalent to creativity itself. Measures of creative potential explain only part of the variation in creative achievement, which also depends on actual creative engagement, expertise, environmental opportunities, developmental experiences, and domain-specific factors Benedek, (2024). Conclusions can also vary according to how divergent-thinking responses are selected, scored, and aggregated (Saretzki et al., 2025). These methodological differences reinforce the need to distinguish performance on a particular task from creativity as a broader phenomenon.

Generation and evaluation are also more closely connected than traditional stage models sometimes suggest. Creative work does not necessarily begin with unrestricted generation and end with a separate phase of convergent selection. Evaluation can occur throughout the process, influencing which ideas are pursued, modified, or abandoned and thereby shaping subsequent ideation. Research on idea evaluation suggests that successful evaluation depends on both individual expertise and openness to alternative possibilities, as well as on the demands of the task and context (Rietzschel et al., 2023). The processes involved in generating an idea, recognizing its potential, refining it, and turning it into a viable outcome may therefore differ, and the same individual characteristics need not support each process equally.

Insight offers another example of why creativity cannot be reduced to a single cognitive mechanism. Insight solutions are often experienced as sudden, but this subjective experience does not mean that the solution emerges without prior processing. Changes in problem representation, memory activation, unsuccessful search, and incubation can all precede the conscious emergence of an insight (Kounios and Beeman, 2014). Moreover, an insight still needs to be evaluated and integrated with task requirements. Insight is therefore better understood as one possible route to a creative solution rather than as a distinct or exclusively spontaneous creative mechanism.

Creative cognition also extends beyond processes occurring “in the head.” Writing, sketching, manipulating objects, moving through physical space, interacting with others, and using symbolic or technological tools can all shape how a problem is represented and explored. External representations may reduce memory demands, make relationships easier to perceive, reveal constraints, or suggest possibilities that would be difficult to generate through mental simulation alone. Research on distributed and embodied cognition has long emphasized that cognitive activity can be supported by interactions among individuals, artifacts, and environments (Clark and Chalmers, 1998; Hutchins, 1995). In creativity research, sociocultural accounts similarly emphasize the role of artifacts, material environments, and affordances in creative activity (Glăveanu, 2013, 2020). The distinction between psychological and environmental explanations is therefore not absolute: changing the material or technological environment can change the cognitive operations available to an individual, while knowledge and goals influence which environmental possibilities become relevant.

Personality characteristics should be interpreted with similar caution. Openness to experience is one of the most consistent personality correlates of creativity and creative achievement (Puryear et al., 2017). Curiosity, imagination, and willingness to engage with novelty may increase the likelihood of exploring unusual possibilities. However, openness alone does not guarantee creative behavior or achievement. Whether these tendencies are translated into creative activity also depends on domain knowledge, motivation, persistence, opportunities for action, and environmental support. More broadly, creative potential, creative behavior, and creative achievement should not be treated as interchangeable outcomes. (Benedek 2024) argues that translating potential into achievement depends partly on actual creative engagement and on developmental and environmental conditions. A personality characteristic may therefore encourage exploration without necessarily predicting whether that exploration will result in socially recognized creative achievement.

Motivation provides another example of why simple directional claims can be misleading. Intrinsic motivation has long been linked to creativity because interest and enjoyment can support exploration, persistence, and sustained engagement with challenging problems (Amabile and Pratt, 2016). However, extrinsic motivation is not necessarily detrimental. Rewards can reduce creativity when they are experienced as controlling, but under other conditions they may direct attention toward creative goals, provide information about competence, or operate alongside intrinsic motivation (Amabile and Pratt, 2016; Cerasoli et al., 2014). The relevant question is therefore not simply whether motivation is intrinsic or extrinsic, but how motivational states interact with autonomy, reward structures, expertise, personal goals, and task demands. Motivation contributes to creativity partly by influencing whether cognitive resources are engaged and sustained, but its effects depend on the conditions in which that engagement occurs.

Emotion shows a similar pattern. Positive affect has often been associated with broader attention and greater associative flexibility, whereas some negative emotional states have been linked to persistence or more analytical forms of processing. However, broad distinctions between positive and negative affect can obscure differences among specific emotions, arousal levels, regulatory goals, personality characteristics, creative domains, and outcome measures. Ivcevic (2022), for example, argues that conclusions about emotion and creativity depend strongly on whether studies assess divergent thinking, self-perceived creativity, creative behavior, or achievement. Instrumental accounts of emotion regulation similarly suggest that an emotional state can be useful when it supports the individual's goals, even when its valence is negative (Leung et al., 2022).

Recent experimental evidence illustrates this conditional relationship. Zhu et al. (2023) found that cognitive reappraisal facilitated creativity particularly among individuals lower in openness to experience, with cognitive flexibility contributing to this effect. This does not mean that reappraisal universally increases creativity. Rather, it shows how an emotion-regulation strategy can interact with another individual characteristic and become more useful under particular conditions. The relationship between emotion and creativity may therefore depend less on whether an emotion is simply positive or negative than on how it is regulated, what type of processing it promotes, and whether that processing fits the demands of the current creative activity.

These findings also raise an important methodological issue. Divergent-thinking performance, creative self-beliefs, everyday creative behavior, domain-specific products, and recognized creative achievement capture different aspects of creativity and operate over different timescales (Benedek, 2024; Ivcevic, 2022). A variable associated with originality in a brief laboratory task may therefore have little relation to sustained creative behavior, just as a characteristic associated with lifetime creative achievement may not predict moment-to-moment idea generation. Some apparently contradictory findings may result from treating these different outcomes as interchangeable. Greater conceptual and measurement specificity is therefore necessary when drawing conclusions about the psychological factors associated with creativity.

Overall, psychological research does not identify a single cognitive capacity, motivational state, personality profile, or emotional condition that reliably produces creativity. Instead, it points to a set of resources whose relevance changes across individuals, tasks, stages, domains, and contexts. From this perspective, psychological processes matter through their coordination with one another and with embodied capacities, developmental histories, material environments, social relationships, and technological systems. Creative cognition can therefore be understood as a form of adaptive regulation in which generation, evaluation, motivation, emotion, and metacognitive monitoring are coordinated in response to changing goals and constraints.

## 4 Social ecologies of creativity: culture, education, organizations, and technologies

Creativity does not develop independently of the social environments in which people live and act. Ideas are generated, expressed, evaluated, circulated, and recognized within cultural, institutional, relational, material, and increasingly technological contexts. Systems and sociocultural approaches have therefore challenged explanations centered exclusively on individual capacities, showing that creativity acquires meaning through relations among individuals, audiences, domains, artifacts, norms, and systems of recognition (Csikszentmihalyi, 1999; Glăveanu, 2013, 2020). From the perspective proposed here, however, context is more than an external influence that facilitates or inhibits individual creativity. Social environments help shape the possibilities people encounter, the forms of novelty they recognize, the risks they are willing to take, and the outcomes that acquire value.

Culture provides a clear example. Creativity is not understood or evaluated in the same way across cultural contexts. Ideas about originality, usefulness, authorship, and innovation vary across societies and historical periods, influencing both how people engage in creative activity and how their products are judged (Sawyer and Henriksen, 2023). Research comparing Western and East Asian contexts, for example, has identified differences in the relative emphasis placed on novelty, individual expression, social appropriateness, usefulness, and continuity with existing traditions. At the same time, recent reviews caution against treating “Western” and “Eastern” cultures as homogeneous categories and emphasize substantial variation both within and between cultural contexts (Chang and Cho, 2023). Cultural influences are therefore better understood as situated values, expectations, and practices than as fixed characteristics of national groups.

This variability also has methodological implications. Creativity measures developed within one cultural or educational tradition cannot automatically be assumed to assess the same phenomenon in another. Divergent-thinking tasks, originality ratings, consensual assessments, and self-report measures may reflect particular assumptions about novelty, individuality, verbal expression, or task engagement. Cross-cultural differences in performance may therefore reflect differences in creative processes, differences in evaluative standards, measurement non-equivalence, or a combination of these factors (Chang and Cho, 2023). Simply translating a creativity test into another language is not sufficient; researchers must also consider whether the task, scoring criteria, and underlying concept of creativity have comparable meanings across contexts.

Language and other symbolic systems further connect cognition and culture. Language is not simply a means of communicating ideas already formed; it provides categories, narratives, and symbolic resources through which possibilities can be represented and shared. Creative activity therefore draws on culturally accumulated knowledge while also recombining, extending, or challenging it (Glăveanu, 2020). Novelty itself is relational: something can be recognized as new only in relation to what a community already knows or expects. Cultural knowledge can therefore both constrain and support creativity. Existing conventions may restrict what appears possible, but they also provide the knowledge and symbolic resources that make reinterpretation and transformation possible.

Multicultural experience illustrates this relationship particularly well. Exposure to different cultural frameworks can broaden the range of knowledge and perspectives available to an individual and has been associated with greater cognitive flexibility and creative performance (Leung et al., 2008; Maddux et al., 2021). However, exposure alone is unlikely to be sufficient. Its effects depend on how people process and integrate cultural experiences, as well as on factors such as openness, identification, language competence, domain knowledge, and the quality of intercultural engagement (Maddux et al., 2021). What appears to matter, therefore, is not simply contact with different cultures, but whether individuals can engage meaningfully with multiple systems of knowledge and representation.

Educational environments provide another example of why context should not be classified simply as supportive or restrictive. A long-standing debate concerns whether formal education limits creativity through standardization, conformity, evaluation, and emphasis on correct answers, or instead provides the knowledge, skills, and disciplinary structures needed for creative development. Recent reviews suggest that this tension remains unresolved (Rawlings and Cutting, 2024). Research on formal schooling also points to several routes through which creativity can be supported, including explicit creativity training, inquiry-based learning, problem solving, classroom climate, teacher practices, and access to material and digital resources (Gu et al., 2026).

From the present perspective, the opposition between structure and freedom is too simple. Structure can limit exploration when it becomes overly rigid or punitive, but it can also provide the knowledge, strategies, feedback, and disciplinary conventions needed to develop an idea. Similarly, autonomy may encourage exploration but may be less useful when learners lack the knowledge or metacognitive skills required to evaluate and refine their ideas. The effects of educational practices therefore depend on how guidance, autonomy, expertise, feedback, assessment, resources, and developmental characteristics are combined. The same teaching practice may support creativity for one learner or at one stage of expertise while being less effective at another.

Children's creativity makes this issue particularly clear. Systems approaches have traditionally emphasized domain knowledge and social or professional validation, raising questions about whether children's productions should be considered creative when they draw on limited expertise or are not recognized by an established field (Csikszentmihalyi, 1999). The present framework approaches the question differently. Rather than asking whether children possess creativity as a general property, it asks how developmental capacities, emerging knowledge, exploratory behavior, educational practices, available materials, and social evaluation combine in particular creative acts. A child's production may be highly novel relative to the child's own previous experience while remaining conventional from the perspective of an established domain. These judgments are not necessarily incompatible; they refer to different levels of creative significance (Benedek, 2024).

The same principle applies in organizations. Research has identified autonomy, supervisory support, psychological safety, collaboration, and access to resources as conditions that can support creativity. However, contemporary organizational research increasingly emphasizes interactions between individual characteristics and working-environment characteristics rather than universal contextual effects (Zhou and Hoever, 2023). Actor–context relationships can be synergistic, compensatory, inhibitory, or characterized by diminishing returns. A supportive environment may strengthen the contribution of particular individual resources, compensate for their absence, or provide little additional benefit when those resources are already strong.

Psychological safety illustrates why these relationships are not straightforward. When individuals feel able to propose uncertain ideas, disagree, experiment, and acknowledge mistakes without excessive interpersonal risk, they may be more willing to participate, exchange information, and contribute ideas (Edmondson and Lei, 2014; Frazier et al., 2017). Yet psychological safety is neither necessary nor sufficient for creative achievement. Its effects depend on leadership, expertise, team composition, task demands, motivational climate, and performance pressures (Zhou and Hoever, 2023). Creative work can also involve criticism, disagreement, competition, and constraint. The more useful question, therefore, is not whether supportive environments produce creativity, but when particular forms of support or pressure become helpful or harmful for particular people and tasks.

This also explains why creative achievement can occur in environments characterized by competition, scarcity, hierarchy, or demanding leadership. Such examples do not show that support is irrelevant; rather, they show that contextual factors do not operate independently. Pressure may discourage experimentation in one situation while increasing focus or persistence in another. Similarly, a supportive environment may encourage people to propose ideas without ensuring that those ideas will be original, useful, or successfully implemented. Context influences creativity through its interaction with individual, task, and organizational characteristics.

Organizational creativity is also a multilevel process. Conditions that increase individual idea generation do not necessarily improve collective innovation. Teams must combine different forms of knowledge, coordinate expertise, manage disagreement, select among alternatives, and develop ideas into workable outcomes. Diversity illustrates this tension. Heterogeneous teams can draw on a broader range of information and perspectives, but diversity can also increase coordination difficulties and conflict if differences are not effectively integrated (van Knippenberg and Mell, 2016). Whether diversity supports collective creativity therefore depends on communication, leadership, task interdependence, psychological safety, and processes for integrating different perspectives (Zhou and Hoever, 2023). Creativity in organizations is consequently better understood as emerging from interactions across individual, team, and organizational levels.

These multilevel relationships are especially visible in digital environments. Search engines, recommendation systems, social media, collaborative platforms, and other digital infrastructures shape what information people encounter, whom they interact with, which ideas become visible, and how creative products circulate. Technology therefore does more than provide a channel for expressing creativity. By organizing access, attention, feedback, and visibility, digital systems become part of the environment in which creative possibilities are generated and selected.

Networked technologies can expand opportunities for creative collaboration by making it easier to exchange information, work across geographical boundaries, access expertise, and participate in collective production. At the same time, platform architectures and recommendation systems influence what users see and which forms of content receive attention. Research on algorithmic recommendation has shown that these systems can shape patterns of exposure and cultural consumption, sometimes increasing access to diverse material while also reinforcing particular patterns of attention and popularity (Bakshy et al., 2015; Chaney et al., 2018). Engagement metrics can similarly reward content that is immediately recognizable or salient. Digital systems may therefore expand the overall range of available information while narrowing the subset repeatedly presented to particular users. Their implications for creativity depend on the interaction among platform design, user behavior, social networks, and economic incentives.

Generative artificial intelligence makes the role of technology even more direct. AI systems can now participate in ideation, recombination, evaluation, and production rather than simply providing access to information. They may generate alternatives, externalize partially formed ideas, support iteration, or reduce technical barriers to implementation (Ivcevic and Grandinetti, 2024). However, the effects of these tools depend on how they are used. Expertise, prompting strategies, prior knowledge, evaluation skills, task demands, and reliance on generated outputs can all shape the resulting creative process.

Recent evidence shows why these effects must be considered at multiple levels. Doshi and Hauser (2024) found that access to generative AI improved evaluations of individual creative stories, particularly among less creative writers, but also increased similarity across outputs and reduced collective diversity. The same technology can therefore benefit individual performance while constraining diversity at the collective level. More broadly, a recent meta-analysis of human–AI collaboration found substantial variation in outcomes, with benefits depending on the relative strengths of humans and AI and on how their contributions were combined (Vaccaro et al., 2024). Technology therefore does not have an inherently positive or negative effect on creativity; its consequences depend on the configuration of users, tasks, system capabilities, and interaction conditions.

Generative AI also blurs traditional distinctions between creator, tool, environment, and audience. AI systems draw on large bodies of existing cultural material, generate possibilities that shape subsequent human choices, and operate within platforms that affect how outputs circulate and are evaluated. Creative activity can therefore involve recursive relationships among individual cognition, collective cultural knowledge, computational systems, institutions, and audiences. Treating technology simply as another external environmental variable would miss this more active role within contemporary creative processes.

Taken together, research on culture, education, organizations, and technology does not identify social conditions that universally promote or inhibit creativity. Instead, it shows that environments provide different opportunities, constraints, resources, and evaluation systems whose effects vary across individuals, tasks, domains, developmental stages, and levels of analysis. Context is therefore not simply the setting in which creativity occurs; it helps shape what forms of creative action become possible and which outcomes acquire value. From the perspective proposed here, understanding creativity requires examining how social and technological environments interact with individual capacities, psychological processes, and developmental histories rather than searching for universally “creative” environments.

## 5 Discussion: from creative potential to sociotechnical ecologies

The argument developed throughout this Perspective challenges linear and monocausal explanations of creativity. Neurobiological characteristics, cognitive abilities, personality, motivation, social environments, cultural systems, and technological resources can all contribute to creative activity, but none is inherently creative. Their influence depends on how they combine with other processes for particular individuals, activities, and developmental, social, and historical conditions. The proposed framework therefore does not simply claim that creativity has multiple determinants. Rather, creativity is best understood through the changing configuration of factors that enable, constrain, and influence one another over time.

This position builds on systemic, sociocultural, and interactionist approaches while extending their scope. Csikszentmihalyi's (1999) systems model showed that creativity cannot be located exclusively within the individual, because creative products acquire significance through relations among person, domain, and field. Glăveanu (2013, 2020) similarly moved beyond individualistic explanations by emphasizing relations among actors, actions, artifacts, audiences, and affordances and by integrating psychological, social, and material dimensions. More recent interactionist approaches have focused on Person × Task × Situation dynamics (Tromp and Sternberg, 2022) and on the interplay between individual characteristics and contextual conditions (Zhou and Hoever, 2023). The present framework builds on these relational perspectives but extends them across levels: psychological and sociocultural processes are also embodied, shaped by development and history, and increasingly mediated by technologies that influence how ideas are generated, selected, circulated, and evaluated.

The distinctive claim is therefore not that biology, mind, society, and technology should simply be added to a longer list of determinants. Instead, these processes are treated as analytically distinguishable but functionally interdependent. Their effects may reinforce, compensate for, interfere with, or even operate in opposite directions at different levels. A condition that supports one component of creativity may constrain another; a resource that benefits one person may have little effect on another; and an intervention that improves individual performance may reduce diversity at the collective level. Creativity is therefore better understood as a configurational phenomenon than as the cumulative result of independent influences.

### 5.1 Cross-level mechanisms and testable propositions

A configurational account must also explain how processes at different levels connect. The framework identifies four broad mechanisms through which this can occur: constraint, affordance, regulation, and feedback/selection. These mechanisms are neither exhaustive nor mutually exclusive. Instead, they describe recurring ways in which biological, psychological, social, cultural, and technological processes can influence one another over time.

Constraints define or limit the possibilities available at a particular moment. Biological capacities, existing knowledge, task requirements, cultural conventions, institutional rules, material resources, and algorithmic architectures can all restrict what actions or representations are accessible. Yet constraints are not necessarily detrimental to creativity. They can limit exploration, but they can also reduce an unmanageable search space, focus attention, or encourage a new representation of a problem. Research on creative constraints similarly suggests that constraints can either hinder or support creativity depending on their nature and the conditions under which they operate (Acar et al., 2019). Their effect therefore depends on the relationship between the constraint, the individual, the task, and the stage of the creative process.

Affordances refer to possibilities for action that arise through the relationship between an individual and the environment. Knowledge, tools, collaborators, materials, cultural resources, and technologies can make certain actions possible without determining whether those possibilities will actually be recognized or used (Glăveanu, 2020). An AI system, for example, may enable rapid generation of alternatives, but the usefulness of this affordance depends on the user's expertise, goals, evaluative skills, and interaction strategies. Affordances are therefore relational: they emerge from the interaction between the resources an environment provides and the capacities of the person acting within it.

Regulation refers to how people explore, monitor, select, maintain, revise, or abandon available possibilities. At the individual level, metacognitive monitoring, cognitive control, motivation, and emotion contribute to this process (Lebuda and Benedek, 2023). At interpersonal and institutional levels, feedback, norms, incentives, collaboration, and evaluation can perform related selective functions. Regulation connects levels because internally generated ideas are continuously adjusted in response to external demands, while external resources become useful only through the goals, knowledge, and regulatory capacities of individuals and groups.

Finally, feedback and selection make creative activity recursive and developmental. Ideas receive feedback, products are accepted or rejected, strategies succeed or fail, and cultural or technological systems amplify some outputs while overlooking others. These responses can alter later knowledge, motivation, expectations, opportunities, and standards of evaluation. Over longer periods, successful creative products may themselves modify domains, institutions, cultural conventions, or technological systems. Cross-level interaction is therefore not a one-way causal chain from biology to society, or from environment to individual cognition. It is an ongoing cycle in which constraints and affordances shape available possibilities, regulation guides action, and feedback changes the conditions under which future creative activity occurs.

This account leads to several testable propositions that distinguish the framework from the general claim that “everything interacts.” First, the configurational-dependence proposition predicts that the effect of a creativity-related factor will vary according to the conditions in which it operates. Cognitive flexibility, autonomy, social support, or AI assistance, for example, should have differentand potentially opposite effects depending on task demands, expertise, developmental characteristics, and other resources or constraints present.

Second, the temporal-specificity proposition predicts that factors supporting one phase of creative activity will not necessarily support another. Broad exploration or reduced cognitive constraint may facilitate initial idea generation but become less useful during refinement. Conversely, evaluation and cognitive control may restrict early exploration but become increasingly important during selection and implementation. Process-sensitive and longitudinal studies should therefore reveal changes in the effects of the same factor as creative activity develops over time.

Third, the cross-level divergence proposition predicts that the same intervention can have different or even opposite effects at different levels. A technological, educational, or organizational intervention may improve individual creative performance while reducing collective diversity, or increase idea generation without improving implementation or social recognition. The individual–collective trade-off observed in AI-assisted creativity (Doshi and Hauser, 2024) provides one empirical illustration of this proposition.

Fourth, the developmental-reconfiguration proposition predicts that the effects of the same opportunity or constraint will change as individuals and their environments change. Increases in knowledge, expertise, cognitive development, or technological familiarity should alter which possibilities people can recognize and use and which constraints become important. The same educational, social, or technological condition should therefore not be expected to have identical effects across developmental stages or levels of expertise.

These propositions are empirically testable. If major creativity-related factors consistently showed the same effects across tasks, phases, levels of analysis, and developmental conditions, this would challenge the framework's central assumptions. By contrast, systematic moderation, changes over time, cross-level trade-offs, and developmental differences would support a configurational account. The framework's value therefore lies not in explaining any result after the fact, but in directing research toward specific patterns of conditionality and change.

### 5.2 Contradiction as theoretically informative

This perspective also offers a different way of interpreting conflicting findings in creativity research. Cognitive control has been associated with both higher and lower creative performance; positive and negative affect can each support creativity under different conditions; structure may restrict exploration but also provide knowledge and useful constraints; and supportive environments can encourage creative engagement even though creative achievement can also emerge under competition, scarcity, hierarchy, or pressure. Similarly, influential accounts of cooperation between default-mode and executive networks coexist with recent critiques of attempts to identify a common neural architecture for highly diverse creative activities (Abraham, 2025a; Dietrich, 2024).

Such inconsistencies should not automatically be dismissed as methodological noise or taken to mean that researchers have simply failed to identify the correct determinant of creativity. Some undoubtedly result from differences in measurement, sampling, statistical power, or operationalisation. Others, however, may reflect the conditional nature of creativity itself. If creativity depends on combinations of factors rather than isolated variables, stable main effects should not always be expected across individuals, tasks, domains, developmental periods, and contexts. Cognitive control, for example, may help when a task requires refinement but be less useful during broad exploration. Similarly, the effects of autonomy or social support may depend on expertise, self-regulation, task demands, and interpersonal conditions.

Greater precision is also needed in defining the outcome being explained. Divergent-thinking performance, creative potential, everyday creative behavior, domain-specific products, and socially recognized creative achievement are related but distinct phenomena (Benedek, 2024; Ivcevic, 2022). Abraham (2025b) similarly questions whether the standard originality-plus-usefulness definition adequately captures both the evaluator's perspective and the creator's experience. A variable associated with originality in a short laboratory task should therefore not automatically be expected to predict sustained creative behavior or recognized achievement. Some apparent contradictions may arise because findings from different stages or levels of creativity are treated as though they refer to the same phenomenon.

The framework consequently shifts the empirical question from *Does X increase creativity?* to a more conditional one: *For whom does X matter, at which stage of creative activity, in combination with which other resources or constraints, at which level of analysis, and over what period of time?* These questions provide a basis for deriving conditional rather than universally directional hypotheses.

### 5.3 An illustrative application: rethinking creativity in childhood

Children's creativity provides a useful example of how this framework changes the way a familiar theoretical problem is approached. From a systems perspective, Csikszentmihalyi (1999) argued that culturally significant creativity requires domain knowledge and validation by a field. Young children have relatively limited disciplinary knowledge, and their productions rarely transform established domains or receive professional recognition. From this perspective, children's original productions may be developmentally important without necessarily representing creativity in the same sense as recognized innovation.

The present framework does not require rejecting this distinction. Instead, it changes the question. Rather than asking whether a child is “creative,” it asks what form of creativity is being considered and which combination of developmental, cognitive, social, and environmental conditions makes it possible. A child's exploratory behavior may be supported by curiosity, developmental plasticity, relatively unconstrained representations, and opportunities for play, while being limited by domain knowledge, executive resources, technical skills, or access to cultural tools. Educational guidance may restrict exploration when evaluation is introduced too early, but it can also expand creative possibilities by providing knowledge, strategies, materials, and representational resources.

Social recognition adds another level. A production can be original relative to the child's own experience and meaningful within a classroom or family while remaining conventional from the perspective of a professional domain. These judgments are not necessarily incompatible because they refer to different forms and levels of creative significance. Distinguishing creative potential, behavior, and achievement is therefore particularly important in developmental contexts (Benedek, 2024).

The same example shows why neither knowledge nor freedom is inherently beneficial for creativity. Knowledge provides the resources required for sophisticated innovation but can also strengthen established ways of representing a problem. Freedom can encourage exploration but may provide too little structure for an idea to be developed effectively. Recent reviews similarly suggest that the relationship between education and creativity cannot be reduced to a simple contrast between restrictive instruction and creative freedom (Rawlings and Cutting, 2024). What matters is how knowledge, exploration, guidance, motivation, cognitive development, evaluation, and available resources work together at a particular stage of development.

This reasoning also applies beyond childhood. The “lone genius” model is insufficient not because individual abilities are unimportant, but because exceptional creative achievement cannot be explained by individual ability or environmental opportunity alone. People exposed to similar cultural or technological environments can follow very different creative trajectories because they bring different knowledge, motivations, cognitive resources, dispositions, relationships, and developmental histories to those environments. Similarly, a highly supportive cultural environment alone cannot explain why only some individuals produce transformative work. The relevant question is how individual resources and environmental opportunities interact and reinforce one another over time.

### 5.4 Methodological implications: from main effects to configurations

A configurational theory also requires changes in research design and operationalisation. Controlled laboratory paradigms remain valuable because they allow researchers to isolate particular processes and test causal relationships. The present framework does not reject experimental methods or divergent-thinking tasks. It does, however, caution against generalizing effects observed in highly constrained tasks to creativity as a whole. Different measures capture different processes, outcomes, and timescales (Abraham, 2025a; Dietrich, 2024; Ivcevic, 2022).

The framework therefore does not require a single new measure of creativity. Instead, select measures based on the level, phase, and timescale of the phenomenon being studied. Divergent-thinking tasks can assess particular aspects of idea generation; product ratings can evaluate characteristics of creative outputs; behavioral measures can capture engagement in creative activities; and measures of achievement or recognition can address longer-term social outcomes. When the research question spans levels, measurement should also span those levels—for example, by combining cognitive or affective measures with task characteristics, social interactions, environmental resources, or technological trace data.

Future research should combine experimental precision with methods that capture change across contexts and time. Longitudinal and intensive repeated-measures designs can examine how creative behavior changes as motivation, knowledge, relationships, and environments evolve. Experience-sampling and ambulatory approaches can capture creative cognition in everyday contexts (Rominger et al., 2022). Multilevel models can distinguish within-person from between-person variation and test whether the same factor has different effects under different conditions. Social-network methods can examine how ideas move between people, while digital trace data can show how creative products are generated, modified, shared, and selected within technological environments.

Interactions should therefore not be treated simply as secondary statistical qualifications of main effects. In many cases, they are the phenomenon that the theory seeks to explain. Studies could test, for example, whether cognitive flexibility is more strongly related to originality when tasks require representational change; whether expertise moderates the effects of autonomy or AI assistance; whether psychological safety influences idea generation differently from idea selection; or whether the same technology produces different outcomes at individual and collective levels. Person-centered and configurational approaches may also help identify different combinations of characteristics that lead to similar creative outcomes.

Ecological validity also requires greater attention to time. Creative work often develops through extended cycles of generation, failure, feedback, revision, collaboration, and selection. Factors that support the beginning of a creative process may not be equally useful later. Exploration may be advantageous during early ideation but costly during refinement; evaluation may initially restrict exploration but later become essential; and external feedback may redirect a project in ways whose importance becomes clear only over time. Research designs should therefore capture transitions, feedback loops, and changing relationships rather than only static associations.

### 5.5 Implications for education and organizations

The same principles have practical implications. Fostering creativity does not simply mean maximizing factors that are positively associated with creative performance. In education, neither unrestricted freedom nor highly structured instruction provides a universal solution. Effective learning environments likely require different combinations of knowledge, autonomy, challenge, feedback, collaboration, resources, and opportunities for exploration, depending on learners' developmental and domain-specific needs. Recent reviews similarly suggest that creativity in formal education can be supported through several pathways, including explicit instruction, pedagogical practices, classroom climate, and contextual resources (Rawlings and Cutting, 2024; Gu et al., 2026).

This also has implications for creativity training. Training a general, decontextualised “creative ability” may transfer poorly if learners do not also develop the capacity to recognize when particular strategies are useful, regulate generation and evaluation, adapt to domain constraints, and make effective use of available resources. Metacognitive skills may be particularly important because they help individuals monitor and adjust their strategies as task demands change (Lebuda and Benedek, 2023). Creativity education should therefore involve not only generating ideas but also learning how to coordinate exploration, knowledge, evaluation, motivation, emotion, collaboration, and tools.

Similar caution is needed in organizations. Psychological safety, autonomy, interdisciplinary collaboration, diversity, and access to resources can support creativity, but none guarantees it. Actor–context research shows that individual and contextual factors can interact in synergistic, compensatory, inhibitory, or diminishing ways (Zhou and Hoever, 2023). Organizational design should therefore focus less on identifying universally “creative environments” and more on understanding how leadership, expertise, incentives, team composition, task demands, communication, and resources work together. Disagreement, constraint, competition, or time pressure may support creativity under some conditions while reducing exploration or participation under others.

In terms of the mechanisms proposed here, organizational interventions change the affordances, constraints, regulatory processes, and feedback available to employees. Greater autonomy expands the range of possible actions, but its usefulness depends on expertise, motivation, self-regulation, task ambiguity, and available resources. Psychological safety can reduce the interpersonal costs of proposing uncertain ideas, while evaluation and reward systems influence which ideas are selected, developed, or abandoned. Organizations therefore influence creativity not simply by being more or less supportive, but by shaping the possibilities available for action and the feedback through which ideas develop.

### 5.6 Creativity in human–AI systems

Generative artificial intelligence provides a particularly clear example of why creativity needs to be analyzed across levels. AI can increase the number of available ideas, support recombination, reduce technical barriers, and provide rapid feedback. Yet this does not mean that AI universally increases creativity. Its effects depend on user expertise, task characteristics, interaction strategies, system capabilities, evaluation skills, and the stage of the creative process at which it is used (Ivcevic and Grandinetti, 2024).

Within the proposed framework, AI can function simultaneously as an affordance, a constraint, a regulatory resource, and a source of feedback. It creates new possibilities by rapidly generating alternatives, externalizing partially developed ideas, or making technical operations more accessible. At the same time, it can constrain the range of possibilities through the data, statistical regularities, and system architectures that shape its outputs. AI can also support regulation by helping users compare, evaluate, and revise ideas, while its responses influence subsequent choices and expectations. Which of these functions becomes most important depends on the interaction between the user, the task, and the technological system rather than on the technology alone.

Recent evidence illustrates that the creative consequences of generative AI depend not simply on access to the technology, but on how human and technological resources are configured within the creative process. Chen and Chan (2024), for example, compared expert and non-expert users completing a creative advertising task with large language models used either as “ghostwriters,” taking the primary role in content generation, or as “sounding boards,” providing feedback on human-generated material. The effects differed substantially across configurations. Using the LLM as a sounding board improved outcomes for non-experts, whereas relying on it as a ghostwriter provided no comparable advantage and was detrimental to expert users. Their analyses further suggested that ghostwriting could produce an anchoring effect, constraining subsequent human contributions. Thus, the same technological capability functioned differently depending on both user expertise and the role assigned to AI within the task.

A similar pattern emerges when the structure of human–AI interaction itself is manipulated. McGuire et al. (2024) found that participants produced less creative poetry when they were given an AI-generated poem to edit than when they wrote independently. However, this disadvantage disappeared when the system was redesigned to support iterative co-creation, with human and AI alternately contributing to the developing poem. The co-creative configuration also preserved higher creative self-efficacy, which helped explain differences in creative performance. These findings suggest that AI assistance cannot be adequately characterized as a single intervention: its effects depend partly on how technological affordances are organized, how human agency is preserved, and how these arrangements interact with users' psychological resources.

Together, these studies illustrate why human–AI creativity is better understood as a configurational rather than additive phenomenon. AI may operate as an affordance by expanding the possibilities available to creators, yet become a constraint when generated outputs anchor subsequent thinking or displace processes that users would otherwise regulate themselves. The relevant unit of explanation is therefore not AI assistance itself, but the configuration of human capacities, technological functions, task demands, and interaction structures through which that assistance operates.

Evidence beyond individual studies supports this broader interpretation. A systematic review and meta-analysis by Vaccaro et al. (2024) found substantial variation in human–AI performance, with outcomes depending on the relative capabilities of humans and AI and on how their contributions were combined. Although these findings extend beyond creativity specifically, they reinforce the need to examine human–AI performance relationally rather than treating technological assistance as having a fixed effect. In creative contexts, the relevant question is therefore not simply whether AI enhances creativity, but under which human–task–technology configurations it does so.

AI also blurs the boundary between creator, tool, and environment. Generative systems draw on large bodies of existing cultural material, produce outputs that influence subsequent human choices, and operate within platforms and institutions that shape how creative products circulate and are evaluated. Human–AI creativity can therefore be understood as a sociotechnical process in which human knowledge and goals, algorithmic outputs, interface characteristics, cultural data, and systems of evaluation jointly influence what is generated and what is ultimately recognized as creative.

Importantly, these configurations are not static. As people become more familiar with generative AI, they may develop new prompting strategies, evaluative skills, expectations, and ways of integrating AI-generated material into their work. The same technological affordance may therefore have different effects for novice and experienced users or at different stages of adoption. At a broader level, widespread use of generative systems may also alter creative practices and cultural expectations concerning novelty, originality, and authorship. Human–AI creativity should consequently be studied not only in terms of immediate performance gains but also through its longer-term effects on users, practices, and the cultural environments in which creativity is produced and evaluated.

From this perspective, AI is neither inherently creativity-enhancing nor creativity-reducing. Its creative significance emerges from changing relationships among technological capabilities, human resources, task demands, interaction structures, social practices, and systems of evaluation. Human–AI creativity therefore provides a particularly clear illustration of the broader argument developed in this Perspective: the contribution of any resource depends on the configuration in which it operates, the level and stage at which its effects are assessed, and how those relationships change over time.

## 6 Conclusion

The framework developed in this Perspective moves beyond both the idea of creativity as an individual capacity and the search for factors that universally enhance or inhibit creative performance. Creative activity emerges from changing relationships among biological capacities, psychological processes, developmental histories, social and cultural environments, and technological systems. None of these factors is inherently creative; its contribution depends on how it interacts with the other resources and constraints present at a particular time.

The main theoretical proposal is therefore a shift in the unit of explanation, from isolated determinants to dynamic configurations. Constraint, affordance, regulation, and feedback/selection describe recurring ways in which processes operating at different levels can influence one another over time. These mechanisms do not imply that all levels are equally important in every creative act. Rather, they offer a way to examine how particular combinations of individual and contextual factors become relevant under specific conditions.

This perspective also generates testable expectations. The same factor should not necessarily have the same effect across individuals, tasks, stages of the creative process, or developmental periods; factors that support idea generation may not support evaluation or implementation; and benefits at the individual level may coexist with costs at the collective level. Evidence of consistently stable effects across these conditions would challenge the configurational account, whereas systematic moderation, changes over time, and cross-level trade-offs would support it.

More broadly, this framework changes the question that creativity research should ask. Rather than identifying *what makes people creative* in general, research should examine *which combinations of people, processes, environments, and technologies support or constrain creativity, for whom, under which conditions, at which stage and level, and over time*. Addressing this question will require greater integration across disciplines and methods, together with research designs capable of following creative processes as they develop in real-world contexts. Creativity can thus be understood not as a fixed property located within individuals, but as an embodied, developmental, relational, and sociotechnical process that emerges within changing environments.
